# Association between varicose veins anatomical pattern and procedural complications following endovascular laser photothermolysis for chronic venous insufficiency

**DOI:** 10.1590/1414-431X20198330

**Published:** 2019-04-08

**Authors:** C. Molnar, D. Opincariu, T. Benedek, M. Toma, C. Nicolescu

**Affiliations:** 1 1st Surgery Clinic, University of Medicine and Pharmacy of Tîrgu Mureş, Tîrgu Mureş, Romania; 2 TopMed Medical Center, Tîrgu Mureş, Romania; 3 Department of Anatomy, University of Medicine and Pharmacy of Tîrgu Mureş, Tîrgu Mureş, Romania; 4 Clinic of Cardiology, University of Medicine and Pharmacy of Tîrgu Mureş, Tîrgu Mureş, Romania; 5 Center of Advanced Research in Multimodality Cardiovascular Imaging, CardioMed Medical Center, Tîrgu Mureş, Romania; 6 Faculty of Medicine, University of Medicine and Pharmacy of Tîrgu Mureş, Tîrgu Mureş, Romania

**Keywords:** Varicose veins, Venous insufficiency, Laser phlebectomy, Truncal veins

## Abstract

We sought to assess clinical characteristics and pattern of collateral network involvement associated with development of truncal (systematized) versus diffuse/non-truncal (non-systematized) varicose veins (VVs) in patients undergoing endovascular laser photothermolysis for chronic venous insufficiency (CVI). Secondly, we aimed to assess whether the type of VVs influenced the procedural complications of endovascular laser therapy. A total of 508 patients with hydrostatic VVs of the lower limbs who underwent endovenous laser treatment were included, out of which 84.1% (n=427) had truncal VVs (group 1) and 15.9% (n=81) had diffuse (non-systematized) VVs (group 2). Patients with truncal varices were significantly older (47.50±12.80 *vs* 43.15±11.75 years, P=0.004) and those with associated connective tissue disorders were more prone to present diffuse VVs (P=0.004). Patients in group 1 presented a significantly higher number of Cockett 1 (P=0.0017), Cockett 2 (P=0.0137), Sherman (P<0.0001), and Hunter (P=0.0011) perforator veins compared to group 2, who presented a higher incidence of Kosinski perforators (P<0.0001). There were no significant differences regarding postoperative complications: thrombophlebitis (P=0.773), local inflammation (P=0.471), pain (P=0.243), paresthesia (P=1.000), or burning sensation (P=0.632). Patients with more advanced CEAP (clinical, etiologic, anatomic, pathophysiologic) classes were older (P<0.0001), more were males (39.05 *vs* 27.77%, P=0.0084), more were prone to present ulcers (P<0.0001) and local hyperthermia (P=0.019), and presented for endovenous phlebectomy after a longer time from symptom onset. In patients with CVI, systematized VVs were associated with a more severe clinical status and a distinct anatomical pattern of perforators network compared to non-systematized VVs, which is more common in advanced stages.

## Introduction

Chronic venous insufficiency (CVI) is a common vascular disorder affecting almost one third of the adult population, having a substantial negative impact on healthcare costs and on the health-related quality of life (1–3). The prevalence of CVI and varicose veins (VVs) has been shown to vary across geographic locations and between genders, being estimated at approximately 10–15% in male subjects, and around 20–25% in females (4). Besides female gender, other risk factors for CVI include older age, pregnancy, family history of venous disorders, obesity, and professional environments that require prolonged orthostasis (5,6).

The pathophysiological mechanism of CVI and VVs consists mainly in alteration of venous return, triggered by the incompetence of venous valves or by an intrinsic defect of the vein wall. The hemodynamic changes caused by altered venous return can lead to stasis, blood pooling, and increased venous pressure in the lower limbs (7,8). Perforator veins, that connect the deep and superficial venous networks, play an important role in the initiation and progression of varicose vein dilatation, as the presence of reflux in the perforator veins leads to increased venous pressure in the superficial venous network, with secondary varicose dilation of superficial veins (9,10).

The onset of superficial CVI occurs due to a vertical venous reflux caused by the ostial venous valve at the level of the saphenous-femoral junction, or due to a horizontal reflux triggered by the degradation of the ostial valves of the perforator veins that leads to venous reflux from the profound to the superficial venous network.

In most cases, CVI is caused by the presence of the vertical venous reflux caused by the malfunction of either the sapheno-femoral or sapheno-popliteal venous valve. In these situations, the reflux towards the great and small saphenous veins will lead to venous dilations and blood pooling in the perforator veins. If the truncal, profound venous system is intact, with a proper venous return due to the calf and thigh muscular pump, the venous circulation at the level of the perforators will not be disturbed. However, prolonged orthostatic positions with a lack of muscle contraction in the lower limbs will lead to an alteration of the profound venous return, thus triggering an exertion in the ostial valves and the appearance of a horizontal venous reflux from the profound towards the superficial venous system.

The therapeutic option of advanced CVI is surgical treatment, including various techniques such as surgical excision of VVs with ligation and stripping of the great or superficial saphenous vein, sclerotherapy, phlebectomy via multiple minor incisions, or endovascular therapy using radiofrequency or laser, as minimally invasive alternatives to surgery. An efficient surgical therapy in CVI leads to reversal of the superficial venous reflux, which will cause decongestion of the profound or truncal venous system.

Truncal veins (or systematized veins) include the great and small saphenous veins, the anterior accessory saphenous vein, and the Giacomini vein (intersaphenous). The non-systematized veins include the non-truncal superficial venous system, which is located subcutaneously and has a reticular appearance. Treatment of varicose veins includes ligation or exclusion of the perforator veins. Perforator veins are venous branches that connect the truncal and non-truncal venous systems, in a number of up to 150 in each lower limb. The nomenclature of perforator veins categorizes them according to the served anatomical region, but they are also named by the physician who described them. The Cockett 1, 2, and 3 (superior, medium, and inferior) perforator veins are located in the inferior two-thirds of the lower limbs; the Sherman perforating vein includes the upper tibial perforating veins, that connect the posterior arch vein with the posterior tibial veins; the Hunter perforator, also known as the adductor canal perforator, is located in the distal third region of the thigh, however, rarely in the adductor canal; the Dodd perforator is also located in the adductor canal in the mid-thigh region; the Boyd perforator vein, also named the paratibial perforating vein, is located in the initial part of the calf, below the knee joint (11).

While the role of collateral networks in progression of CVI is well established, there is no study so far on the association between severity of clinical symptoms and the degree of collateral network involvement in patients with different types of VVs. At the same time, the association between systematized (truncal) or non-systematized (diffuse) vein dilatation and clinical severity or procedural outcomes following surgical correction of VVs is still unknown.

The aim of this study was to compare clinical characteristics, severity, and pattern of collateral network involvement associated with development of truncal (systematized) versus diffuse (non-systematized) VVs in a large series of patients undergoing endovascular laser photothermolysis for CVI in a single center. As a secondary objective, we aimed to assess whether the type of VVs could influence the procedural outcomes of minimally invasive endovascular laser therapy.

## Material and Methods

This was a single center prospective observational study conducted in the TopMed Medical Center of Tîrgu Mureş, Romania, between 2010 and 2017 and included 508 consecutive patients aged over 18 years, with hydrostatic VVs of the lower limbs. The study was approved by the local ethics committee of TopMed Medical Center, all patients gave informed consent for the study procedures, and the study was conducted in accordance with the principles stated in the Declaration of Helsinki. Patients with deep vein thrombosis associated with lower limb ischemia or those who did not offer informed consent were excluded from the study.

### Study population

The study population was divided into two groups: group 1 included 427 patients with systematized VVs, represented by truncal or valvular varicose veins, and group 2 included 81 patients with non-systemized VVs, representing diffuse varicose disorders, affecting the collateral venous system.

### Study procedures

The following data were collected from the medical records of all patients included in the study: age, gender, associated diseases, clinical severity, days of hospitalization, time from symptoms onset to presentation, surgical technique, and postoperative complications (venous ulcers, local hyperthermia, and heaviness in the lower limb).

A venous Doppler ultrasound examination was performed in all the patients prior to the surgical intervention in order to mark the trajectory of the superficial veins, as well as the communicant venous system, and all information regarding the local anatomy, the presence or absence of a collateral venous network, and the type of perforator veins was collected. Doppler ultrasound of the lower limb venous system offered essential information on venous anatomy, compressibility, presence of reflux, and assessment of flow augmentation. A saphenous vein is a direct branch that belongs to the great or small saphenous vein system. A non-saphenous vein is an indirect branch of this saphenous system or a collateral vein. The reflux in the perforator vein was diagnosed by the presence of a reverse flow on Doppler ultrasound lasting more than 0.5 s and a variable diameter of the vein. For example, the diameter of the calf perforator vein was between 2 and 3.5 mm and the thigh perforators had diameters between 3 and 3.5 mm on average.

All the enrolled patients underwent phlebectomy with the use of endovenous selective laser photothermolysis.

### Surgical technique

Endovenous laser treatment was performed in all the patients using the Biolitec equipment (Biolitec, Inc, USA), with a diode radial laser attached to a core fiber.

The surgical technique used for selective phlebectomy followed the 4-step approach. The first step included ligature of the external or internal saphenous arch, with cannulation of the incipient collector of the internal or external saphenous vein, respectively. The second phase included photo-thermo-coagulation by application of laser energy in the internal or external saphenous trunks, while sealing the vertical reflux. The third and fourth steps included endovenous laser ablation of the collateral veins located in the calf and thigh and directly within the affected perforator veins. After venous sclerosis and closure, the entire laser fiber was removed from the vein and intradermal sutures were placed at the incision site, followed by application of a sterile wound dressing.

### Statistical analysis

All statistical analyses were performed using the InStat Graph Pad software (USA). Fisher's exact test (or Student’s *t*-test for age) was used to compare the baseline characteristics of patients in group 1 and group 2. Continuous values are reported as means±SD, and statistical significance was determined using the Mann-Whitney test. Statistical significance was set at an alpha of less than 0.05.

## Results

### Clinical characteristics of the study population

From the 508 patients included in the study, 84.1% (n=427) presented truncal (systematized) varicose veins (group 1), and 15.9% (n=81) presented diffuse (non-systematized) varicose veins (group 2).

Clinical characteristics of the study population are presented in Table 1. Analysis of sex distribution showed that both systematized and non-systematized VVs were more frequent in females, affected in 65.5% of cases in group 1 and in 96.1% of cases in group 2. At the same time, patients with truncal varices were significantly older compared to patients with diffuse varicose veins (group 1: 47.50±12.80 *vs* group 2: 43.15±11.75 years old, P=0.004).

**Table 1:** Baseline characteristics of the study population and CEAP (clinical, etiologic, anatomic, pathophysiologic) classification.

Clinical characteristic	Group 1 (n=427)	Group 2 (n=81)	P value
Age (mean±SD)	47.5±12.8	43.1±11.7	0.04
Gender, female, n (%)	267 (62.52%)	69 (85.18%)	<0.0001
Diabetes, n (%)	14 (3.27%)	0 (0%)	0.1410
Comorbidities, n (%)			
Arterial hypertension	110 (25.76%)	20 (24.69%)	0.8397
Obesity	59 (13.81%)	10 (12.34%)	0.8600
Ischemic heart disease	10 (2.43%)	2 (2.46%)	1.0000
Surgical disorders affecting the connective tissue*	14 (3.27%)	9 (11.11%)	0.0048
Clinical symptoms, n (%)			
Discomfort	256 (59.95%)	51 (62.96%)	0.6115
Paresthesia	22 (5.15%)	3 (3.70%)	0.7815
Lower limb heaviness	273 (63.93%)	29 (35.80%)	<0.0001
Cramps	149 (34.89%)	26 (32.09%)	0.6273
Edema	390 (91.33%)	74 (91.35%)	0.8347
Local hyperthermia	312 (73.06%)	42 (51.85%)	0.0001
Pruritus	295 (69.08%)	51 (62.96%)	0.2783
Venous ulcers	39 (9.13%)	2 (2.46%)	0.0446
CEAP classification, n (%)			
C2	3 (0.70%)	8 (9.87%)	<0.0001
C3	170 (39.81%)	53 (65.43%)	<0.0001
C4	215 (50.35%)	18 (22.22%)	<0.0001
C5	20 (4.68%)	0 (0%)	0.0559
C6	13 (3.04%)	0 (0%)	0.2389

^*^Including abdominal hernias, incisional hernias, hemorrhoids. Group 1: truncal varicose veins: Group 2: diffuse varicose veins. CEAP classes: C2: varicose veins, distinguished from reticular veins by a diameter of 3 mm or more; C3: edema; C4: changes in skin and subcutaneous tissue secondary to cardiovascular disease, such as pigmentation or eczema, lipodermatosclerosis or atrophie blanche; C5: healed venous ulcer; C6: active venous ulcer. Fisher's exact test or *t*-test was used for statistical analyses.

There was no significant difference between the two groups regarding the presence of diabetes (P=0.140), arterial hypertension (P=0.839), obesity (P=0.860), or ischemic cardiac disease (P=1.000). However, we found that patients with associated connective tissue disorders such as abdominal hernias, incisional hernias, or hemorrhoidal disease were more prone to present diffuse varicose veins (P=0.004).

There was no difference between the two groups regarding the bilateral involvement of the lower limbs (43.09% in group 1 *vs* 48.14% in group 2, P=0.4005).

### Anatomical distribution

**Figure 1:**
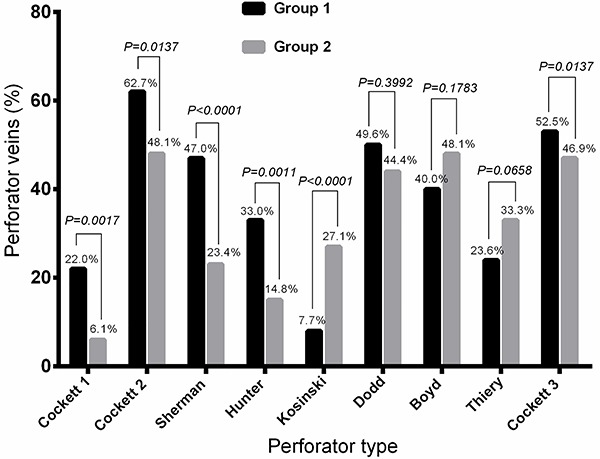
Anatomical distribution of the perforator veins. Data are reported as means±SD. Group 1: truncal varicose veins: Group 2: diffuse varicose veins. Fisher's exact test was used for statistical analyses.

The anatomical distribution of the perforator veins is presented in Figure 1. Patients in group 1 presented a significantly higher number of Cockett 1 (P=0.0017), Cockett 2 (P=0.0137), Sherman (P<0.0001), and Hunter (P=0.0011) perforator veins compared to group 2, which presented a higher incidence of Kosinski perforator veins (P<0.0001).

### Clinical severity

The most frequent symptom was lower limb edema for both groups (91.33 *vs* 91.35%, P=0.834), however patients in group 1 presented more frequent lower limb heaviness (P<0.0001), local hyperthermia (P=0.0001), and varicose ulcers (0.044) (Table 1).

Clinical severity of the VVs was assessed based on CEAP (clinical, etiologic, anatomic, pathophysiologic) classification, which showed that patients with truncal VVs presented a more advanced CEAP class compared to patients with diffuse VVs (Table 1).

At the same time, a sub-analysis of clinical characteristics in relation to CEAP class showed that patients with more severe clinical presentation, classified in CEAP class C4, C5, and C6, were significantly older (P<0.0001), more frequently males (39.05 *vs* 27.77%, P=0.0084), more prone to present ulcers (P<0.0001) and local hyperthermia (P=0.019), and presented for endovenous phlebectomy after a longer time from the onset of symptoms compared to those in CEAP class C1, C2 or C3 (Table 2).

**Table 2:** Comparative analysis according to the CEAP (clinical, etiologic, anatomic, pathophysiologic) classification of the study population.

Clinical and anatomical characteristics	CEAP C2, C3 (n=234)	CEAP C4, C5, C6 (n=274)	P value
Age, years (mean±SD)	40.82±11.57	51.91±11.39	<0.0001
Males, n (%)	65 (27.77%)	107 (39.05%)	0.0084
Females, n (%)	169 (72.22%)	167 (60.94%)	0.0084
Truncal varicose veins, n (%)	173 (73.9%)	248 (90.5%)	<0.0001
Diffuse varicose veins, n (%)	61 (26.1%)	18 (9.5%)	<0.0001
Time from onset to presentation, days (mean±SD)	9.53±7.11	16.39±8.60	<0.0001
Days of hospitalization (mean±SD)	1.70±0.59	1.62±0.64	0.0690
Heaviness in the lower limbs, n (%)	130 (55.55%)	172 (62.77%)	0.0986
Local hyperthermia, n (%)	151 (64.52%)	203 (74.08%)	0.0195
Venous ulcers, n (%)	0 (0%)	41 (14.96%)	<0.0001
Surgical disorders of the connective tissue (hernias, incisional hernias, hemorrhoids), n (%)	8 (3.41%)	14 (5.10%)	0.4749
Cockett 1 perforator, n (%)	32 (13.67%)	67 (24.45%)	0.0022
Cockett 2 perforator, n (%)	127 (54.27%)	180 (65.69%)	0.0074
Sherman perforator, n (%)	87 (37.17%)	133 (48.54%)	0.0100
Kosinski perforator, n (%)	29 (12.39%)	26 (9.48%)	0.2937
Hunter perforator, n (%)	57 (24.35%)	96 (35.03%)	0.0089

CEAP classes: C2: varicose veins, distinguished from reticular veins by a diameter of 3 mm or more; C3: edema; C4: changes in skin and subcutaneous tissue secondary to cardiovascular disease, such as pigmentation or eczema, lipodermatosclerosis or atrophie blanche; C5: healed venous ulcer; C6: active venous ulcer. Fisher's exact test was used for statistical analyses.

On the other hand, analysis of the anatomical distribution of the perforator veins showed that patients in lower CEAP categories (C2 and C3) showed a lower incidence of perforator veins compared to patients with more severe venous insufficiency, independent on the type of perforator vein (Cockett 1, Cockett 2, Sherman, Kosinski or Hunter) (Table 2).

**Figure 2:**
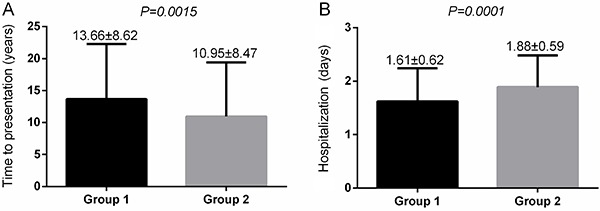
**A**, Time from onset of symptoms to presentation in patients with truncal versus (Group 1) and diffuse varicose veins (Group 2). **B**, Number of hospitalization days of patients according to groups. Data are reported as means±SD. Fisher's exact test was used for statistical analyses.

Interestingly, patients with truncal VVs presented longer time intervals from the onset of symptoms to presentation (13.66±8.62 months *vs* 10.95 ± 8.47 months, P=0.0015) but a lower number of hospitalization days (1.61±0.62 days *vs* 1.88±0.59 days, P=0.0001) compared to patients with diffuse VVs (Figure 2).

### Post-procedural complications

There was no significant difference between the two groups in relation to postoperative complications, which were recorded in similar rates in both groups: thrombophlebitis (9.13 *vs* 7.4%, P=0.773), local inflammation (24.4 *vs* 19.75%, P=0.471), local pain (7.72 *vs* 3.70%, P=0.243), ecchymosis (2.81 *vs* 4.93%, P=0.500), paresthesia (2.34 *vs* 1.23%, P=1.000), pigmentation (14.28 *vs* 17.28%, P=0.485), or burning sensation (18.26 *vs* 16.04%, P=0.632). Pulmonary embolism, as the most severe possible complication of chronic venous insufficiency, was not encountered in any of the included patients.

The most common post-procedural complications included ecchymosis, postoperative pain, and induration, which were usually mild and were treated with local application of heparinated gels with anti-inflammatory, analgesic, antithrombotic, and anti-lymphedema effects. Superficial thrombophlebitis and deep venous thrombosis were treated with low molecular weight heparin and novel oral anticoagulants. All patients benefited from postoperative administration of sulodexide (250 LSU) 2 cp/day. As for the Kosinski's perforators, we encountered hypoesthesia on the superficial peroneal nerve territory and, in extremely rare cases, on the deep peroneal nerve territory. In the thermal ablation treatment of the small saphenous vein we encountered damage to the medial sural cutaneous nerve and exceptionally to the lateral cutaneous nerve. The usage of a radial fiber and the reduction of the energy dose decreased postoperative redoubtable pain.

## Discussion

In this study, we demonstrated that there were significant differences in clinical presentation and anatomical distribution of the perforator network in patients with systematized versus non-systematized VVs. However, these differences did not have any impact on procedural outcomes following endovascular laser therapy, as demonstrated by a comparable incidence of post-procedural complications in both groups.

Systematized VVs seemed to be associated with a significantly higher disease severity compared to diffuse VVs, as indicated by more severe clinical manifestations, a higher incidence of varicose ulcers, and a significantly more advanced CEAP class. At the same time, patients with truncal VVs were older but did not have a significantly higher rate of comorbidities compared to those with diffuse VVs.

It is well known that females present a higher risk for CVI. Interestingly, our study indicated that female gender was associated with a higher incidence but a lower severity of CVI compared to male gender. Diffuse VVs, which seem to be more frequent in female gender, were associated with clinical symptoms of lower severity (venous ulcer, local hyperthermia, and heaviness) and with less advanced CEAP classes compared to truncal VVs.

Clinical presentation of CVI includes the presence of enlarged dilated veins in the lower limbs, skin trophic modifications related to vein malfunction including pigmentation, dermatitis, lipodermatosclerosis, atrophy, edema, and venous ulcers. The symptoms comprise pain, congestion, irritation, local hyperthermia, cramps, heaviness, swellings, and itching in the lower limbs, (12–14). Initially seen as a cosmetic problem, VVs can lead to a significant decrease in the quality of life, causing venous insufficiency with increased discomfort, and, in more advanced stages, even limb amputation or death (15,16). The CEAP classification is recommended by the current guidelines for assessment of the clinical status, origin, anatomy, and pathophysiology of CVI and VVs, while Duplex ultrasonography should be performed as the non-invasive technique of choice in patients with suspected venous disease (17–19).

An interesting finding of our study was that for patients with advanced CEAP (C4, C5, and C6 classes), 90.5% had truncal VVs, which supports the association between truncal VVs and severity. However, it should be noted that patients with systematized disease presented later for surgery compared with those with diffuse disease, which could explain the higher severity of their clinical presentation.

We succeeded to demonstrate that the anatomical pattern of perforator veins is directly associated with the type of VVs. We found certain types of perforator veins (Cockett 1, Cockett 2, Sherman, and Hunter) more frequent in truncal VVs, while Kosinski perforator veins were significantly more frequent in diffuse VVs. As the study indicated that truncal VVs were associated with an increased clinical severity, it seems that certain perforator veins were directly related to the severity of the disease. However, patients with less advanced CEAP classes presented a lower incidence of perforator veins, independent of the type of perforator.

According to the authors’ knowledge, there was no study so far demonstrating the correlation between the type and location of perforator veins and the severity of the disease, and our data could open a new path for other studies in this field.

The purpose of therapies for patients with chronic venous insufficiency is to decompress sources of increased venous pressure; therefore, the initial treatment includes compressive stockings while more advanced treatments include various surgical interventions (20). Advanced treatments include vein stripping, ultrasound guided foam sclerotherapy, and radiofrequency and laser ablation. The latter utilizes thermal energy to injure and contract the venous wall, and has been shown to improve the clinical severity class (shift from CEAP class C2 to C1, persistent venous occlusion, and lower rates of recurrence via venous re-permeabilization). Moreover, laser therapy and stripping were shown to promote significantly higher reflux-free rates at one year compared to foam sclerotherapy (94.2% for laser ablation, 95.2% for stripping, and 83.7% for sclerotherapy, P<0.001) (21). The current European Guidelines for treatment of great and small saphenous vein reflux in patients with clinically significant chronic venous insufficiency recommend endovenous thermal ablation techniques preferably to surgery, as they allow faster recovery, require less days of hospitalization, and have similar success rates compared to surgical stripping (22).

Treatment of VVs is generally applied in subjects who present symptoms and signs of venous insufficiency, including pain, edema, or history of phlebitis with post-thrombotic syndrome, and, in less frequent cases, for cosmetic reasons (17). The pharmacological therapy of VVs comprises the use of venoactive drugs such as diosmin, flavonoids, and hesperidin, which have been shown to reduce swelling, accelerate the healing process of ulcers, and help with the remission of trophic skin changes (17,23,24). However, in a meta-analysis on 44 studies that evaluated the efficacy of phlebotonic medications, Martinez et al. (25) concluded that there is no sufficient evidence supporting their effectiveness in CVI.

Therefore, surgical excision of VVs with ligation and stripping of the great or superficial saphenous vein remains the current standard surgical treatment method in cases with involvement of the great or small saphenous veins (26). According to various authors, sclerotherapy is preferred for smaller veins, as it can be performed in office settings, with fast recovery and less frequent complications, but this procedure is reserved for varices with no truncal reflux (27,28). Ambulatory phlebectomy via multiple minor incisions is used for removal of varicose veins that are tributary to the saphenous veins. Endovascular methods with the use of radiofrequency or laser are minimally invasive alternatives to surgery for the treatment of VVs. The laser method, which we also used in our study, comprises the direct application of laser energy, which ablates the venous endothelium, while not affecting the nerves and the perivenous tissue (29,30).

Defty et al. (31) reported a rate of wound infection in 6.8 to 8.6% and a rate of paresthesia between 11 and 21% for patients undergoing surgical treatment for VVs. The postoperative complications of laser therapy in our study were only minor ones and recorded in similar frequencies in both groups.

In this study, we recorded similar procedural outcomes independent of the type of VVs and the type of perforator network. However, it should be noted that patients with truncal VVs spent less time in the hospital compared to those with diffuse disease, which indicates a fast recovery of this patient population despite a higher clinical severity. At the same time, there was no case of pulmonary embolism, the most severe complication of chronic venous insufficiency and subsequent deep vein thrombosis, which is frequent in patients with varicose veins and chronic venous insufficiency. The presence of acute pulmonary embolism can itself be a life-threatening condition, both in the acute phase, as well as for the long term, either by promoting congestive heart failure or pulmonary arterial hypertension, caused by recurrent thromboembolic events (32,33).

As a limitation, the study does not include any follow-up data that would indicate the association between type of VVs and the rate of recurrence after laser thermoablation. Collection of these data is still ongoing at this moment. More work will be required in order to process follow-up data of this patient cohort.

In conclusion, in patients with CVI, systematized VVs were associated with a more severe clinical status compared to non-systematized VVs, and with a distinct anatomical pattern of perforators network, which is more common in advanced stages. However, these differences do not impact procedural outcomes following endovascular laser therapy.
